# Cholera

**Published:** 1852-08

**Authors:** 


### CHOLERA.

This is the fourth year since cholera made its second appearance upon our continent, having broken out about the same time in New York and New Orleans, in the winter of 1848–'9. The epidemic hitherto has conformed in a remarkable manner to the course which it pursued in its former progress, and if it should continue to do so, this is the last summer that it may be expected. It appeared before in Canada, in 1832, invaded the Eastern States, and spread as far as Cincinnati, St. Louis, Louisville and Lexington in the autumn of that year. The next year its fatal ravages were felt in Kentucky and Tennessee; it lingered until 1835, during which it visited Versailles and some other towns in Kentucky and Tennessee with great violence, and then disappeared. Many of the places which suffered in the former epidemic have thus far escaped, and some which were exempt before have suffered with the present plague. At this time we hear of its irruption in numerous localities where it was prevalent in 1833. During all the season it has appeared, from time to time, in one town and another, generally prevailing for a few days only, and with but slight mortality. The force of the pestilence would seem to be spent. It nowhere manifests the malignity which characterized its first appearance. It is not epidemic in any of our great cities, at the same time that most if not all of them have afforded occasional cases of cholera. There appears to be no tendency to an epidemic. Two or three inmates of a family are attacked and carried off, and there it stops. In this city all through the year, we have had such cases, now and then, but although dysentery has been epidemic and cholera morbus and diarrhea have been unusually prevalent, these complaints have rarely if ever run into cholera; from all which we infer that the epidemic tendency has ceased.

